# Haemodialysis versus peritoneal dialysis in children: an eco-audit

**DOI:** 10.1093/ndt/gfae159

**Published:** 2024-07-13

**Authors:** Marine Makhloufi, Pierre-Jean Cottinet, Bruno Ranchin, Bernard Dureuil, Thomas Loppinet, Daniel Grinberg, Aurélie Portefaix, Justine Bacchetta

**Affiliations:** Pediatric Nephrology Rheumatology Dermatology Unit, Reference Center for Rare Renal Diseases, ORKID and ERK-Net networks, Lyon University Hospital, Bron, France; INSA-Lyon, LGEF, UR682, Lyon University, Villeurbanne, France; Pediatric Nephrology Rheumatology Dermatology Unit, Reference Center for Rare Renal Diseases, ORKID and ERK-Net networks, Lyon University Hospital, Bron, France; Biomedical Department, Hospices Civils de Lyon, France; Clinical Investigation Center P-1407, UMR 5558, LBBE – EMET, CNRS, Hospices Civils de Lyon, Lyon, France; Department of Cardiac Surgery, Hôpital Cardiologique Louis Pradel, Lyon Medical School, Lyon, France; Clinical Investigation Center P-1407, UMR 5558, LBBE – EMET, CNRS, Hospices Civils de Lyon, Lyon, France; Pediatric Nephrology Rheumatology Dermatology Unit, Reference Center for Rare Renal Diseases, ORKID and ERK-Net networks, Lyon University Hospital, Bron, France; INSA-Lyon, LGEF, UR682, Lyon University, Villeurbanne, France; Biomedical Department, Hospices Civils de Lyon, France; Clinical Investigation Center P-1407, UMR 5558, LBBE – EMET, CNRS, Hospices Civils de Lyon, Lyon, France; Department of Cardiac Surgery, Hôpital Cardiologique Louis Pradel, Lyon Medical School, Lyon, France; INSERM 1033 Research Unit, Lyon, France; Lyon Est Medical School, Claude Bernard Lyon 1 University, Lyon, France

To the Editor,

The healthcare sector is one of the biggest producers of greenhouse gases, causing 4.4% of the world net carbon emissions [1]. Extrarenal purification techniques use large quantities of consumables, water and power. The ultimate goal proposed by the Global Environmental Evolution in Nephrology and Kidney Care (GREEN-K) initiative is to provide sustainably powered and produced low-impact net zero waste kidney replacement therapies resilient to climate threats [2]. This requires education, procurement, infrastructure, innovation and sustainable clinical pathways.

The number of children undergoing maintenance dialysis is far less than adults (<1% of the incident dialysis patients), but awareness on environmental impact is also emerging. Data in paediatric dialysis remain scarce, despite paediatric specificities, notably wider use of peritoneal dialysis (PD) [3], care in tertiary centres often far from the patient’s home and a higher weekly average number of haemodialysis (HD) sessions [4]. We aimed to assess the ecological impact of automated PD and HD in maintenance paediatric dialysis.

Environmental data were prospectively collected for all children undergoing maintenance dialysis from 1 to 15 May 2023 in this observational single-centre study. A single physician (M.M.) collected data, being helped by an expert bioengineer (P.J.C.) and a biomedical technician (B.D.). Carbon footprints and water consumption were evaluated separately. To evaluate the carbon footprint of PD and HD, the different steps for those procedures, from the arrival of the products to their archiving and destruction (end of life), were considered, as illustrated in the (Annex 1 and Supplementary Figs. 1 and 2) and as previously described [5]. The system studied was delimited (system boundary) and included all the steps of PD and HD. The elements not integrated in this system are considered as outside the system boundary, e.g. the electricity consumption of the building or the carbon footprint for the transportation of staff members. Retrospective collection of individual medical data was approved by the local institutional review board (session 07/17/2023-23-5150): age, initial disease, duration and frequency of dialysis sessions, frequency of follow-up in outpatient clinics in PD, dialysate/substitution flow rates during HD sessions, distance from the home/hospital. Patients were divided into three groups: PD, HD with central venous catheter (CVC) and HD with arteriovenous fistula (AVF). Quantitative data are described as median [interquartile range (IQR)]. Non-parametric Kruskall–Wallis and Mann–Whitney tests were performed, using R statistical software (R Foundation for Statistical Computing, Vienna, Austria).

Fifteen children were undergoing maintenance dialysis, at a median age of 10.2 years (IQR 12.8): five underwent PD, four HD-CVC and six HD-AVF. The weekly number of HD sessions was 4 (range 3.0–5.0) for HD-AVF and 6 (range 5.8–6) for HD-CVC. In PD, seven sessions per week were performed in all patients; outpatient clinics occurred every 21 days (range 15–21). The median duration of sessions was 11.2 h (IQR 9.1–11.3) in PD, 4.0 h (IQR 4.0–4.4) in HD-AVF and 3.0 h (IQR 3.0–3.3) in HD-CVC. PD and HD patients travelled a median of 65 km/week (IQR 52.7–119) and 249 km/week (IQR 128.2–471), respectively (*P* = .04). Relevant characteristics of the patients are summarized in Supplementary Table 1. The weight of waste collected during a session of HD-CVC averaged 2.1 kg (IQR 1.8–2.4) compared with 1.0 kg (IQR 0.9–1.1) in HD-AVF and 1.4 kg (IQR 1.3–1.5) in PD (*P* = .01), with a corresponding weight for a year of 608 kg (IQR 491–733), 203 kg (IQR 160–263) and 511 kg (IQR 476–548) per patient.

As illustrated in Fig. 1, annual carbon dioxide (CO_2_) equivalent emissions were 6.3 tons (IQR 3.9–11.4) in HD and 4.6 tons (IQR 3.9–4.8) in PD (*P* = NS); annual water consummation was 359 020 l (IQR 234 850–444 380) in HD and 185 236 l (IQR 180 120–208 960) in PD (*P* = .019). Annual CO_2_ equivalent emissions were 11.1 tons (IQR 10.0–13.0), 3.9 tons (IQR 2.8–4.2) and 4.6 tons (IQR 3.9–4.8) for HD-CVC, HD-AVF and PD, respectively (*P* = .041), while annual water consumption was 463 880 l (IQR 439 330–471 840), 267 640 l (IQR 201 400–334 760) and 185 236 l (180 120–208 960), respectively (*P* = .01). Patients undergoing HD-CVC had more frequent sessions of shorter duration than patients on HD-AVF. Therefore, annual water consumption in HD-CVC was multiplied by 1.7 compared with HD-AVF (mainly explained by the higher frequency of dialysis in the HD-CVC group). Although performed daily, PD remained the least energy-consuming technique. Patients’ transportation was the predominant category in terms of CO_2_ equivalent emissions in HD (75% for HD-AVF and 61% for HD-CVC), while consumables represented 58% of CO_2_ equivalent emissions in PD. CO_2_ production due to consumables, cardboard and information leaflets were 2.5 times more important in HD-CVC than in HD-AVF. Water consumption due to consumables, cardboard and information leaflets were 1.5 times more important in HD-CVC than in HD-AVF. This analysis took into account monthly disinfection of the HD circuit (requiring 3 m^3^), nightly consumption for softeners regeneration (1 m^3^) and specific consumption of osmosis units (production capacity of 250 l/h, conversion rate ≈60%, production from 6 a.m. to 10 p.m., 6/7). Otherwise, the treatment plan is in automatic rinse mode and circulates 200 l/h, with very little loss. Altogether, this represents an estimated consumption of 884 l for an HD-AVF session and 913 l for an HD-CVC session.

**Figure 1:**
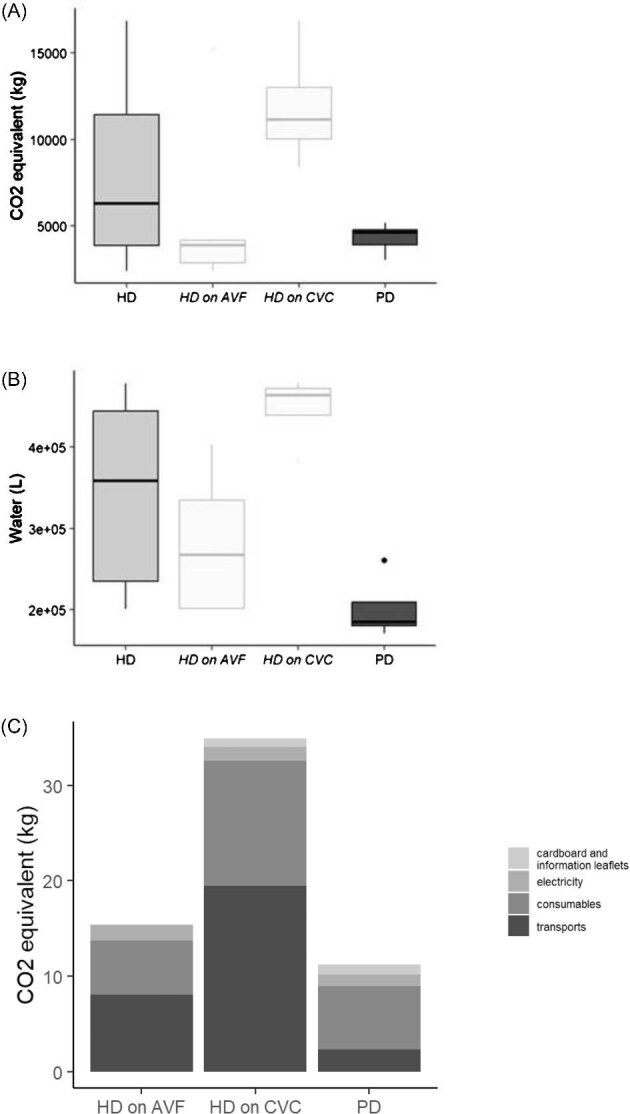
Annual extrapolation of the **(A**) average CO_2_ equivalent emission per patient, **(B)** average water consumption and **(C)** causes of CO_2_ equivalent emission depending on the dialysis technique. Note that the extreme value in the HD-AVF group (A) is represented by the patient travelling the greatest distance to the HD centre: 356 km/session. *P < .05 when comparing annual water consumption between the PD and HD groups; annual water consumption between the PD, HD-CVC and HD-AVF groups; and annual CO_2_ equivalent emissions between the PD, HD-CVC and HD-AVF groups. HDF: haemodiafiltration.

HD-CVC represents on average per patient and per year the equivalent of 9.4 round trip Paris–New York flights for carbon impact [6], and the equivalent of 48 830 dishwashers for water consumption [7]. These numbers obviously question our practice, but should also be presented with caution, especially to patients and families, to avoid the culpability burden of being sick and damaging Mother Earth. These results support what we have been proposing for years to children who cannot receive pre-emptive transplant, namely a proactive approach to PD, for the child's quality of life, preservation of residual renal function and vascular preservation in the long term [8]. The Kidney Disease Outcomes Quality Initiative guidelines reiterate the superiority of AVF over CVC for infections, thrombotic complications, central vein preservation and dialysis efficiency [9]. However, in the smallest children, AVF creation is impossible [10], justifying CVC use. The frequency of sessions is also higher in young children, due to required fluid intake to meet nutritional needs, which requires additional consumables. The scarcity of tertiary paediatric centres also implies that transportation accounts for a larger proportion of greenhouse gas emissions. Patients cannot use public transportation or grouped transport: unfortunately, aside from trying to promote electric cars, we currently have little room for adaptation. If we could avoid the carbon impact of transportation, HD-AVF could even be the least ‘polluting’ technique.

Thus, this is one of the first assessments of the ecological impact of paediatric dialysis highlighting that PD is more than likely the most eco-responsible technique in paediatrics.

## Supplementary Material

gfae159_Supplemental_File

## Data Availability

The datasets generated during and/or analysed during the current study are not publicly available due to confidential data but are available from the corresponding author upon reasonable request.
