# Supplementary material for: Haemodialysis versus peritoneal dialysis in children: an eco-audit
Source: Nephrol Dial Transplant. 2024 Jul 13;39(11):1927–9. doi: 10.1093/ndt/gfae159 (PMC11522548; doi:10.1093/ndt/gfae159)
Supplement: gfae159_Supplemental_File [file gfae159_supplemental_file.pdf]

Supplementary Materials

Annex 1 **2-3**

Supplementary Tables **4-7**

Supplementary Figures **8**

Bibliography of supplemental materials **9**

Annex 1- Materials and Methodology

Study Design

This observational single-centre study was conducted in a tertiary pediatric nephrology unit. The environmental data were prospectively collected for all children below 18 years of age undergoing maintenance dialysis between May 1st and 15th, 2023. A single physician (MM) was involved in data collection, with the help of an expert bioengineer (PJC) and a biomedical technician (BD).

Functional units used in the present study

The present study reports on two functional units for which the carbon footprints and water consumption were separately evaluated: PD and HD.

General concept

To evaluate the carbon footprint of PD or HD, the different steps for those procedures, from the arrival of the products to their archiving and destruction (end-of-life), were considered as illustrated in **Figure 1**. The system studied was delimited (system boundary) and included all the steps of PD and HD. The elements not integrated in this system are considered as outside the system boundary, e.g., the electricity consumption of building or the carbon footprint for the mode of transport of the staff members.

Collection of emission factors for green house emission (GHE) assessment

For each procedure previously selected, data were prospectively collected by two medical staff (MM and BD). The emission factors determined for materials, packaging, and drugs was performed by an independent team of scientists (PJC, MQL) from the LGEF Research Laboratory. GHE was expressed as kg CO_2e and/or water consumption in Liters for an easier comparison with the carbon lifespan and its global warming potential. The total impact is the sum of each inventory data multiplied by its specific emission factor: total impact = Σ(inventory data × emission factor) as presented in **Figure 2** (1).

For the carbon footprint and water consumption analyses of materials and packaging, the software Ansys Granta EduPack® was used according to the "eco-audit" method proposed by Ashby (2), in order to quantify GHE of a product, from its raw material to final use (3,4). This software allowed for an estimation of the GHE and water consumption throughout each phase of the life cycle assessment (LCA), including material

production, product manufacturing, transport, and end-of-life as displayed in **Figure 2**. The emission factors were multiplied by the mean quantity of materials used per functional unit previously collected (5). Because of the interaction between different medical bodies in the procedures studied, the LCA was broken down into different analyses sub-groups, as previously published (6). The references used for the different elements studied (medical devices, energy consumption and transport) are shown in **Table 1**. Since there is currently no consensus on how to report these environmental analyses, it is important to keep in mind for further comparisons that the GRANTA design systems herein allows us to quantify the CO2 released from PD bags and fluid transportation as well as their boxes; however, the waste recycling process was not quantified (7).

Patients

This part consisting of retrospective collection of individual medical data to improve the description of the cohort was approved by the local IRB (*Comité d'éthique des Hospices Civils de Lyon*, session 07/17/2023, number 23-5150). The following data were collected from the medical reports: age, initial renal disease, dialysis method, duration and frequency of dialysis sessions, frequency of follow-up in our outpatient clinics for PD patients, dialysate flow rate during HD sessions, and distance between home and hospital. Patients were divided into three groups: PD patients, HD patients with central venous catheter (CVC), and HD patients with arteriovenous fistula (AVF).

Statistical analysis

Consumption data were averaged over all patients for both PD and HD. Lifecycle analysis data were calculated by the expert bioengineer (PJC). Quantitative data are described as median (IQ). Quantitative data are described by the median and means with the interquartile range (IQ). In our study, all statistical comparisons were conducted using non-parametric tests. Specifically, comparisons between two groups, namely DP and HD patients, were executed utilizing the Mann-Whitney U test. For the assessment of differences among the three groups, a Kruskal-Wallis rank sum test was employed. The statistical analysis was conducted utilizing R Statistical software (8).

Supplementary Tables

Supplementary Table 1: Description of the various data collected from patients.

Dialysis techniques	*Age (years)*	*Session duration (hours)*	*Frequency of sessions/week*	*Dialysate flow rate (ml/min)*	*Substitution flow rate (ml/min)*	*Frequency of consultations*	*Distance from home to reference centre (km)*
HD via AVF	*15.6*	*5*	*3*	*620*	*70*		*39*
	17.0	*4*	*6*	*560*	*100*		*9.8*
	17.2	*4*	*3*	*600*	*110*		*178*
	9.7	*3,5*	*5*	*600*	*100*		*16*
	7.8	*4,5*	*5*	*500*	*75*		*4.6*
	17.3	*4*	*3*	*600*	*85*		*16*
Median (IQR)	***16 (9-17)***	***4 (4.0-4.4)***	***4 (3-5)***	***600 (545-605)***	***93 (74-103)***		***16 (8.5-33.2)***
HD via CVC	*3.4*	*3*	*6*	*300*	*85*		*22*
	7.8	*3*	*6*	*350*	*75*		*40*
	4.4	*4*	*6*	*300*	*35*		*37*
	3.1	*3*	*5*	*500*	*90*		*98*
Median (IQR)	***4 (3-7)***	***3 (3.0-3.3)***	***6 (5.8-6)***	***325 (300-463)***	***80 (45-89)***		***38.5 (26-84)***
PD	*10.2*	*11.2*	*7*			*1x/15 days*	*119*
	17.2	*9*	*7*			*1x/15 days*	*126*
	14.7	*9.1*	*7*			*1x/month*	*8,8*
	2.5	*11.3*	*7*			*1x/3 weeks*	*79*
	18.6	*11.4*	*7*			*1x/month*	*130*
Median (IQR)	***14.7 (6.4-18.0)***	***11.2 (9.1-11.3)***	***7 (7-7)***			***1x21 days (14-30)***	***93 (44-128) ****

PD: peritoneal dialysis; HD: hemodialysis; HDF: hemodiafiltration; CVC: central venous catheter; AVF: arteriovenous fistula

** For PD, we calculated the distance they traveled to come to the consultation (round trip) and we compare dit to one session (7 sessions per week) to be compared to HD.*

Supplementary Table 2:

(a) Descriptive representation of the different elements analyzed to carry out the assessment of the carbon impact of dialysis (9–15)

Main analysis groups	Secondary analysis groups	Elements studied	References	Equivalence
**Medical devices**		Consumables	Granta Design Software (9) **	
		Cardboard and information leaflets	Pro Carton – Packaging for a Better World (10) **	0.326 g of CO_2 equivalent for the production of 1g of cardboard<br>27.86 g of CO2 equivalent for the production of 1g of paper
**Energy consumption**	Water	Consumables	Granta Design Software (9) **	
		Cardboard and information leaflets	International Water Office (11)	500ml of water for the production of 1g of paper<br>230mL of water to produce 1g of cardboard
		Production of dialysate and substitution fluid in HD	Description in the manuscript (bioengineer)	
		PD fluid	Analysis of Daeseung Kyung (12)	0.0002kg of CO_2 for the production of 5L of PD fluid<br>Water consumption: treatment using demineralised water, no water loss (20)
	Electricity	HD and PD generators, HD dialysate purification room	GREEN IT (13)	1kWh emits 0.1kg of CO_2 equivalent
			ADEME (14)	Health sectors :<br>Heating and hot water: 125kWh/m2<br>Other uses (air conditioning): 70kWh/m2
		Heating, lighting	EDF (15)	
			Pro Carton – Packaging for a Better World (10)	Private sector<br>Consumption of 100m2 house: between 10,600 kWh (after 1975) and 15,000 kWh (before 1975) per year
**Transports**		Kilometers by car	ADEME (14)	0.253kg of CO_2 equivalent emitted per kilometer

* It should be noted that since PD is performed at night for all our patients, we only assessed the electricity consumption of the automated dialyser and the heating. PD: peritoneal dialysis; HD: hemodialysis; EDF: électricité de France (French national provider of power)

** Including material production, product manufacturing, transport of products, and end-of-life.

(b) More detailed representation of consumables analyzed via GRANTA Design software (one patient represented per category)

HD ON AVF	Patient 1	Life cycle analysis										
Product name	Production site	**type of sterilization**	**Masse packaging (g)**	Useful mass (g)	Materials (equ kgCo2)	**Production (equ kgCo2)**	**Transport (equ kgCo2)**	**Total**	quantity per dialysis	**Total (equ Co2)**	Quantity of water for a device (L)	Quantity of water per dialysis (L)
FOLIODRAPE	GERMANY	EO	6	18	0,0498	0,00234	0,00128	0,05342	2	**0,10684**	6,79	13,58
SYLAPLAIE pensensement	FRANCE	EO	0,2	1,2	0,0032	0,0001	0,0002	0,0035	0	**0**	0,357	0
GANT STERILLE LATEX (GAMMEX PI HYBRID)	SRI LANKA	R	9	25	0,079704	0,031488	0,00523	0,116422	2	**0,232844**	12,2	24,4
SERINGUE 5ml					0,0168	0,00661	0,000531	0,023941	**2**	**0,047882**	0,365	0,73
SERINGUE 1mL					0,0139	0,00514	0,000443	0,019483	1	**0,019483**	0,123	0,123
Seringue 10ml					0,0218	0,0086	0,00058	0,03098	**0**	**0**	0,475	0
Seringue 30ml					0,0278	0,011	0,00073	0,03953	**0**	**0**	0,683	0
Compresse non tissé stérilles	France	EO	0,8	40	0,0169	0,0035	0,00333	0,02373	2	**0,04746**	4,018	8,036
BOULE en non tissé stérile	FRANCE	EO	0,8	36	0,0124	0,0028	0,00326	0,01846	1	**0,01846**	3,897	3,897
bibag 5008	FRANCE			83	0,246	0,125	0,00657	0,37757	1	**0,37757**	22,744	22,744
SoftPAC	ITALY			86	0,255	0,16	0,00681	0,42181	1	**0,42181**	20,726	20,726
FX Coral (70)	Allemagne	R		228	0,677	0,344	0,181	1,202	1	**1,202**	76,38	76,38
Fresenius 6008 CAREsed BVM-R	Allemagne	R	29	450	1,47	0,683	0,0356	2,1886	1	**2,1886**	124,61	124,61
Sodium ampoule				0,2	0	0	0	0	1			0
LOVENOX				9				0,091	2	**0,182**	1,4	2,8
Supercath 15G								0,084	**2**	**0,168**	1,33	2,66
Supercath 18G								0,084	**0**	**0**	1,33	0
Haemodia raccord dialyse								0,105	**1**	**0,105**	1,84	1,84
Operstrip (6)				(3g tout compris)				0,073	**1**	**0,073**	2,87	2,87
Tegaderm film				1g (tout compris)				0,018	**2**	**0,036**	1,27	2,54
Mépilex Border 5x5cm				3g				0,073	**1**	**0,073**	2,87	2,87
Mépilex Border 7,5x8,5				4g				0,094	**2**	**0,188**	3,82	7,64
Cathéter 25G (orange)				3g				0,032	**1**	**0,032**	1,33	1,33
Haemotronic				9g (sans emballage)				0,101	**1**	**0,101**	4,23	4,23
Dialine 1x/mois)				53g (+10g emballage)				0,529			23,08	0
Robinet 3 voies								0,149	**0**	**0**	2,23	0
									**Total**	**5,620949**		**324,006**

HD on CVC	Patient 1	Life cycle analysis										
Product name	Production site	**type of sterilization**	**Masse packaging (g)**	Useful mass (g)	Materials (equ kgCo2)	**Production (equ kgCo2)**	**Transport (equ kgCo2)**	**Total**	quantity per dialysis	**Total**	Quantity of water for a device (L)	Quantity of water per dialysis (L)
FOLIODRAPE	GERMANY	EO	6	18	0,0498	0,00234	0,00128	0,05342	0	**0**	6,79	0
Champs bleu stérile (petit) dans kit				10				0,05214	2	**0,10428**	4,85	9,7
Champs bleu stérile moyen dans kit				47				0,2456	2	**0,4912**	24,9	49,8
Emballage bleu Kit				38				0,1878	2	**0,3756**	21,2	42,4
GANT nitrille (classiqu)	FRANCE			1,2				0,1004	4	**0,4016**	1,73	6,92
GANT STERILLE LATEX (GAMMEX PI HYBRID)	SRI LANKA	R	9	25	0,079704	0,031488	0,00523	0,116422	2	**0,232844**	12,2	24,4
SERINGUE 20ml +aigille					0,0683	0,02724	0,02125	0,11679	1	**0,11679**	1,48	1,48
SERINGUE 5ml + aiguille					0,0168	0,00661	0,000531	0,023941	4	**0,095764**	0,365	1,46
Seringue 30ml								0,03953	1	**0,03953**	0,683	0,683
SERINGUE 1mL					0,0139	0,00514	0,000443	0,019483	2	**0,038966**	0,123	0,246
Compresses stériles (1unité) petite								0,0088	4	**0,0352**	0,096	0,384
Compresses stériles (1 unité) moyennes								0,0142	30	**0,426**	0,152	4,56
Compresses stériles (1 unité) grande								0,183	10	**1,83**	0,201	2,01
BOULE en non tissé stérile	FRANCE	EO	0,8	36	0,0124	0,0028	0,00326	0,01846	0	**0**	3,897	0
Compresse non tissé stérilles	France	EO	0,8	40	0,0169	0,0035	0,00333	0,02373	1	**0,02373**	4,018	4,018
HEMODIA MEPITEL FILM (dans kit non utilisé)	Suède	EO	13	5	0,02592	0,01296	0,002145	0,041025	2	**0,08205**	3,612	7,224
bibag 5008	FRANCE			83	0,246	0,125	0,00657	0,37757	2	**0,75514**	22,744	45,488
SoftPAC	ITALY			86	0,255	0,16	0,00681	0,42181	1	**0,42181**	20,726	20,726
FX Coral 40 (filtre)	Allemagne	R		106	0,677	0,344	0,181	1,202	1	**1,202**	76,38	76,38
Charlotte								0,056	2	**0,112**	2,58	5,16
LOVENOX				9				0,091	2	**0,182**	1,4	2,8
BLOUSSE STERILLE	China	EA						2,1946	2	**4,3892**	37,8	75,6
Compact flexibaggle				52	0,145	0,089	0,0187	0,2527	0	**0**	10,92	0
Chlorhexidine 2g/70ml juste bouteille PVC				28	0,0656	0,0316	0,00392	0,10112	1	**0,10112**	6,02	6,02
Life line beta	Allemagne	R	16	250	0,783	0,316	0,00741	1,10641	1	**1,10641**	76,34	76,34
Fresenius medical car original accessory				0,5 (emballage compris)				0,0086	0,25	**0,00215**	0,083	0,02075
Microbox boitier de protection								0,092	2	**0,184**	1,97	3,94
Clamp propylène mauve (dans kit)			10,1					0,105	2	**0,21**	2,03	4,06
Robinet 3 voies								0,149	1	**0,149**	2,23	2,23
cathéter 25G (orange)								0,032	1	0,032	1,33	1,33
Phocytan (10ml)								0,016	2	**0,032**	0,83	1,66
haemotronic								0,101	1	**0,101**	4,23	4,23
Tegaderm film								0,018	0	**0**	1,27	0
Operstrips								0,073	0	0	2,87	0
									**Total**	**13,27**		**481,3**

PD	Patient 1	Life Cycle Analysis										Water (L)
Product name	Production site	Masse packaging (g)	Useful mass (g)	Materials (equ kgCo2)	Production (equ kgCo2)	Transport (equ kgCo2)	Total	quantity per dialysis	Total	quantity of water (L)	Quantity of water per dialysis (L)	Quantity of water (L) (poche)
Sleep Safe Set plus + Safe Lock PD nigh Drainge Set	allemagne	31	343	0,876	0,422	0,0404	1,3384	1	1,3384	82,654	82,654	
Adaptateur Fres DPA > baxter		5	32				0,1683	0,8	0,13464	4,749	3,7992	
Baxter solution pour dialyse	belgique		74	0,189	0,0836	0,00799	0,28059	1	0,28059	13,12	13,12	
bicavera 5L	belgique	32	100	0,201	0,0867	0,00801	0,29571	0	0	26,79	0	0
bicavera 3L							0,2716	2	0,5432	24,12	48,24	6
bag for collecting used dialysate	/		176	0,478	0,234	0,00286	0,71486	4	2,85944	36,08	144,32	
bag for collecting used dialysate	/		176	0,478	0,234	0,00286	0,71486	2	1,42972	36,08	72,16	
Mépilex Border 7,5x8,5			4g				0,094	0,3	0,0282	3,82	1,146	
								Total	**6,61419**		**339,044**	**6**

Supplementary Table 3 : Detailed analysis of averages for one session of equivalent CO_2 (kg) for the three types of dialysis and detailed analysis of averages for one session of water consumption (L) for the three types of dialysis.

	**Consumables (kg eq CO_2)**	***Consumables (kg of waste)***	**Cardboard and information leaflets (kg eq CO_2)**	**Electricity (kg eq CO_2)**	***Electricity (kW)***	**Transport (kg eq CO_2)**	**Total (kg eq CO_2)**
**HD on AVF**	5,6	*1*	0,1	1,5	*16.2*	22	29.2
**HD on CVC**	13,4	*2.1*	0,9	1,4	*14.9*	24,9	40.77
**PD**	6,9	*1.4*	1,3	1	*11*	2,7	11.9

	**Consumables**	**Cardboard and information leaflets**	**Dialysate and substitution fluid**	**Total (Litre of water)**
**HD on AVF**	324	81	883	1289
**HD on CVC**	476	106	928	1510
**PD**	355.5	186	11.5	553

HD: hemodialysis; AVF: arteriovenous fistula; CVC : central venous catheter; PD : peritoneal dialysis.

Supplementary Figures

Supplementary Figure 1: Workflow in the procedure PD and HD

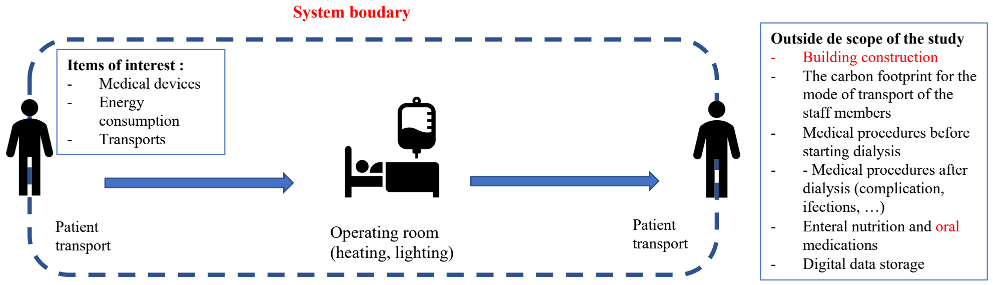

** Medical devices: as exhaustively as possible, we evaluated each waste necessary for dialysis connection and disconnection (compresses, bandages, sterile gloves, connections lines, acid and bicarbonate bag,) in HD and in PD, taking into account* material production, product manufacturing, transport, and end-of-life. *We did not include nitrogen monoxide oxygen mixture (MEOPA) because it was used too discontinuously in HD and in PD (during consultations) to be compared. Also, for a better comparison, we have not taken into account other treatment (like EPO, used in PD and in HD with frequents adaptations).*

Supplementary Figure 2: (a) Conversion of inventory data into equivalent CO_2 emission and/or water waste; (b) Diagram of the Life Cycle Assessment LCA) split in different phases from raw material to final use.

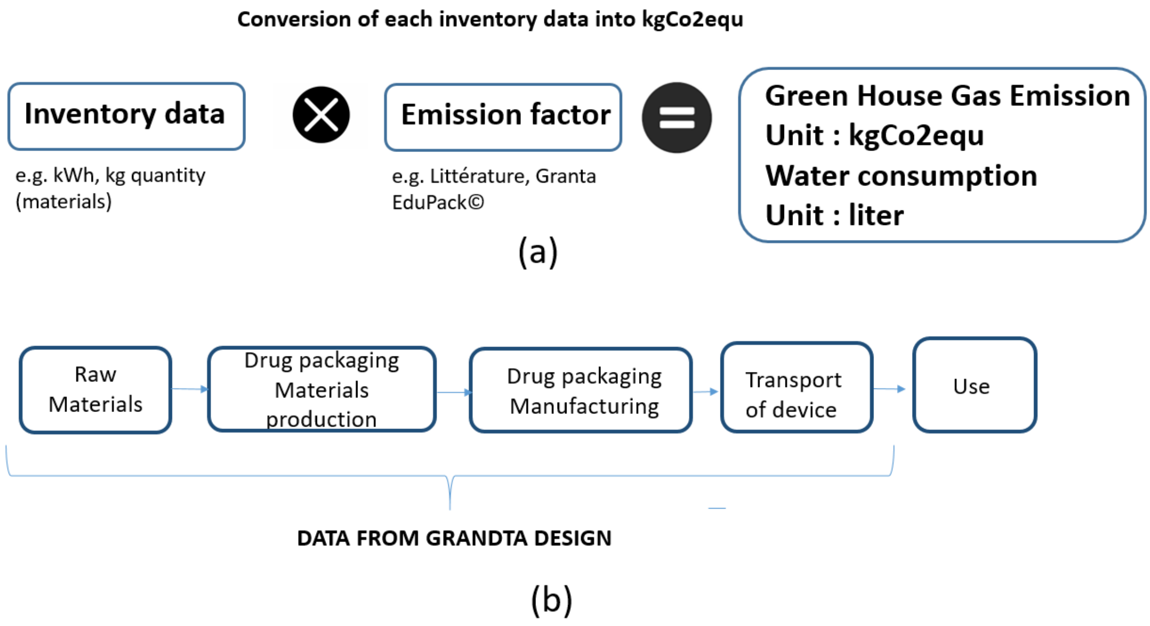

Bibliography of supplementary materials

1. McGinnis S, Johnson-Privitera C, Nunziato JD, Wohlford S. Environmental Life Cycle Assessment in Medical Practice: A User's Guide. Obstet Gynecol Surv. 2021 Jul;76(7):417–28.
2. Ansys Granta: Materials Information Management [Internet]. [cited 2024 Feb 18]. Available from: https://www.ansys.com/fr-fr/products/materials
3. M.F. Ashby Materials and the Environment Oxford, UK, Butterworth Heinemann (2013) - Recherche Google [Internet]. [cited 2024 Feb 18]. Available from: https://www.google.com/search?q=M.F.+Ashby+Materials+and+the+Environment+Oxford%2C+UK%2C+Butterworth+Heinemann+(2013)&rlz=1C5CHFA_enFR1042FR1042&oq=M.F.+Ashby+Materials+and+the+Environment+Oxford%2C+UK%2C+Butterworth+Heinemann+(2013)&gs_lcrp=EgZjaHJvbWUqBggAEEUYOzIGCAAQRRg70gEHMzQzajBqNKgCALACAA&sourceid=chrome&ie=UTF-8
4. Ditac G, Cottinet PJ, Quyen Le M, Grinberg D, Duchateau J, Gardey K, et al. Carbon footprint of atrial fibrillation catheter ablation. Europace. 2023 Feb 16;25(2):331–40.
5. Comparing the environmental impacts of paracetamol dosage forms using life cycle assessment | Environment, Development and Sustainability [Internet]. [cited 2024 Feb 18]. Available from: https://link.springer.com/article/10.1007/s10668-021-01948-2
6. Grinberg D, Buzzi R, Pozzi M, Schweizer R, Capsal JF, Thinot B, Quyen Le M, Obadia JF, Cottinet PJ. Eco-audit of conventional heart surgery procedures. Eur J Cardiothorac Surg. 2021 Dec 1;60(6):1325-1331. doi: 10.1093/ejcts/ezab320. Erratum in: Eur J Cardiothorac Surg. 2021 Oct 05;: PMID: 34411226.
7. McAlister S, Talbot B, Knight J, Blair S, McGain F, McDonald S, Nelson C, Knight R, Barraclough KA. The Carbon Footprint of Peritoneal Dialysis in Australia. J Am Soc Nephrol. 2024 Apr 26. doi: 10.1681/ASN.0000000000000361. Epub ahead of print. PMID: 38671537.
8. R Core Team (2022) R A Language and Environment for Statistical Computing. R Foundation for Statistical Computing, Vienna. - References - Scientific Research Publishing [Internet]. [cited 2023 Nov 27]. Available from: https://www.scirp.org/(S(lz5mqp453ed%20snp55rrgjct55))/reference/referencespapers.aspx?referenceid=3456808
9. GRANTA DESIGN | Dassault Systèmes [Internet]. 2012 [cited 2023 Jun 6]. Available from: https://www.3ds.com/partners/partner-details/200000000040219_GRANTA_DESIGN_LTD
10. Carbon-Footprint-Report-2019-Exec-Summary-French-1.pdf [Internet]. [cited 2023 Jun 11]. Available from: https://www.procarton.com/wp-content/uploads/2019/10/Carbon-Footprint- Report-2019-Exec-Summary-French-1.pdf
11. CNRS. Origine non précisée. http://id.eaufrance.fr/inc/INC00000000000051308; [cited 2023 Jun 11]. Volume d'eau nécessaire pour fabriquer 1 kg de papier. Available from: https://chiffrecle.oieau.fr/627
12. Kyung D, Kim D, Park N, Lee W. Estimation of CO2 emission from water treatment plant – Model development and application. Journal of Environmental Management. 2013 Dec 15;131:74–81.
13. Green IT [Internet]. 2009 [cited 2023 Nov 27]. Combien de CO2 dégage un 1 kWh électrique ? Available from: https://www.greenit.fr/2009/04/24/combien-de-co2-degage-un-1-kwh-electrique/
14. Agence de la transition écologique [Internet]. [cited 2023 Jun 11]. Le saviez-vous ? – Ademe. Available from: https://expertises.ademe.fr/professionnels/entreprises/performance-energetique- energies-renouvelables/dossier/lenergie-hopitaux-cliniques/saviez
15. Tout savoir sur la consommation d'une maison chauffée à l'électricité [Internet]. EDF ENR. [cited2023 Jun 11]. Available from: https://www.edfenr.com/guide-solaire/consommation-moyenne-chauffage-electrique
